# Outreach work in Belgian primary care practices during COVID-19: results from the cross-sectional PRICOV-19 study

**DOI:** 10.1186/s12875-024-02323-6

**Published:** 2024-04-03

**Authors:** Dorien Vanden Bossche, Esther Van Poel, Pierre Vanden Bussche, Benoit Petré, Cécile Ponsar, Peter Decat, Sara Willems

**Affiliations:** 1 Department of Public Health and Primary Care, Faculty of Medicine and Health Sciences, Ghent University, 9000 Ghent, Belgium; 2 Quality and Safety Ghent, Department of Public Health and Primary Care, Ghent University, Ghent, Belgium; 3 Department of Public Health, Faculty of Medicine, University of Liège, Liège, Belgium; 4 Academic Center of Medicine, Institute of Health and Society, UCLouvain, Brussels, Belgium

**Keywords:** Primary healthcare, General practice, Equity, Outreach, COVID-19, PRICOV-19

## Abstract

**Background:**

General practitioners (GPs) have a vital role in reaching out to vulnerable populations during and after the COVID-19 pandemic. Nonetheless, they experience many challenges to fulfill this role. This study aimed to examine associations between practice characteristics, patient population characteristics and the extent of deprivation of practice area on the one hand, and the level of outreach work performed by primary care practices (PCPs) during the COVID-19 pandemic on the other hand.

**Methods:**

Belgian data from the international PRICOV-19 study were analyzed. Data were collected between December 2020 and August 2021 using an online survey in PCPs. Practices were recruited through randomized and convenience sampling. Descriptive statistics and ordinal logistic regression analyses were performed. Four survey questions related to outreach work constitute the outcome variable. The adjusted models included four practice characteristics (practice type, being a teaching practice for GP trainees; the presence of a nurse or a nurse assistant and the presence of a social worker or health promotor), two patient population characteristics (social vulnerability and medical complexity) and an area deprivation index.

**Results:**

Data from 462 respondents were included. First, the factors significantly associated with outreach work in PCPs are the type of PCP (with GPs working in a group performing more outreach work), and the presence of a nurse (assistant), social worker or health promotor. Second, the extent of outreach work done by a PCP is significantly associated with the social vulnerability of the practice’s patient population. This social vulnerability factor, affecting outreach work, differed with the level of medical complexity of the practice’s patient population and with the level of deprivation of the municipality where the practice is situated.

**Conclusions:**

In this study, outreach work in PCPs during the COVID-19 pandemic is facilitated by the group-type cooperation of GPs and by the support of at least one staff member of the disciplines of nursing, social work, or health promotion. These findings suggest that improving the effectiveness of outreach efforts in PCPs requires addressing organizational factors at the practice level. This applies in particular to PCPs having a more socially vulnerable patient population.

**Supplementary Information:**

The online version contains supplementary material available at 10.1186/s12875-024-02323-6.

## Introduction

Outreach work has a long history of engaging with individuals and communities experiencing social exclusion and socioeconomic deprivation [1–3]. This term encompasses a wide range of activities aimed at bridging gaps between users and services. Since its inception in Europe during the early 1980s, this approach has been described by principles of community-centeredness, approachability, participation, and mutual respect in order to support hard-to-reach or hidden populations [4]. Within the field of healthcare, outreach efforts have been primarily directed toward harm reduction related to sexual health, substance abuse, and mental health since the emergence of the HIV and AIDS epidemic in the 1980s [1, 4]. Historically, outreach work has been employed in primary care by different primary care professionals, amongst others community public health nurses, district nurses, and general practitioners (GPs). In recent years, outreach strategies have begun to play a significant role in delivering primary care prevention programs [5]. Numerous primary care (PC) experts view outreach work as a valuable tool in preventing the underutilization of PC services [6–8].

Unfortunately, the integration of outreach work into primary care practices (PCPs) has encountered difficulties due to various competing demands on the primary care professionals working in these PCPs, not in the least on GPs, such as limited time, resources, and practical tools, as well as a lack of incentives [9]. These challenges were further exacerbated during the COVID-19 pandemic [10, 11]. Additionally, outreach work has been described as diverse in its goals, target population, and methods of practice [12]. A recent scoping review revealed considerable variability in the conceptualization of outreach work [13]. This paper defines outreach work as proactive, provider-initiated care that goes beyond the typical care provision driven by patient demand [14, 15]. Numerous studies have demonstrated the effectiveness of outreach work in promoting continuity of care and providing preventive care for diverse health conditions and demographic groups [14, 16–19]. For instance, a telephone outreach program conducted by student volunteers was found to enhance the social wellbeing of nursing home residents during the COVID-19 pandemic [18], while another telephone-based study reported increased adherence to colorectal cancer screening among ethnic minorities [19].

During the COVID-19 pandemic, PCPs had to quickly redesign care delivery to keep up with the ever-evolving information and to optimize care for testing, treatment, and administrative support. At the beginning of the pandemic, due to high workload and physical distancing measures among others, there was a delay in the provision of ‘regular’ care [20, 21]. This resulted in diminished communication with vulnerable populations (including frail elderly, migrants, those with low health literacy or language barriers, victims of domestic violence, homeless populations, and people with a psychiatric history) and inadequate treatment for patients experiencing multiple chronic medical conditions [6]. Moreover, people living in poorer socioeconomic circumstances have higher rates of comorbid chronic health problems, which renders them more susceptible to contracting infections and experiencing severe consequences of the disease compared with others [22, 23]. In addition, the measures to contain the virus’s spread limited social activities, which again induced new health problems that increased the need for care, especially for vulnerable populations [24–26]. Consequently, COVID-19 disproportionately affected vulnerable populations, worsening prevailing inequalities or generating new ones [27, 28].

Public health organizations often attempt to identify and support vulnerable populations on a broad scale. On the other hand, due to their vital role and function in the healthcare system, primary care professionals working in PCPs, for example GPs, hold a unique position that allows them to recognize the most vulnerable patients within their practice. By proactively reaching out to these patients, they can provide education and support to prevent negative health outcomes [7, 29, 30].

This article aimed to generate hypotheses on which factors enabled Belgian PCPs to perform outreach work to vulnerable populations during the COVID-19 pandemic. More specifically, this study aimed to examine associations between practice characteristics, patient population characteristics and the extent of deprivation of practice area on the one hand, and the level of outreach work performed by these PCPs during the COVID-19 pandemic on the other hand. We hypothesized that PCPs with more vulnerable patient populations or situated in more deprived areas, will perform a larger amount of outreaching activities. This is likely due to the higher need for such activities in these areas, which is resource-dependent as well [31]. By modelling these factors, we discuss how the cadre for PCPs may be organized to improve outreach work’s organization in future pandemics. The results may offer a starting point for policy to improve pandemic preparedness to address inequities in healthcare provision. While Belgium has general policies encompassing healthcare coverage and social welfare, specific policies directly enabling PCPs to perform outreach for vulnerable patients might be limited. Existing policies could face limitations such as funding constraints, coordination challenges, regulatory hurdles, and insufficient training or support for GPs. Addressing these limitations and implementing suggested policy improvements could empower PCPs to conduct more effective outreach, reducing healthcare disparities and promoting equity in healthcare access and outcomes for vulnerable patients.

## Materials and methods

The data collection for this study took place in Belgium. The data were collected as part of the PRICOV-19 study to consider how PCPs were organized during the COVID-19 pandemic. The used methodology and measurements in the PRICOV-19 study are already described in detail in another publication by Van Poel et al. [32], summarizing the protocol of the cross-sectional PRICOV-19 study. This multi-country study aimed to describe how GP practices in 38 countries were organized during the COVID-19 pandemic to guarantee safe, effective, patient-centered, and equitable care. The study also seeked to assess the shift in roles and tasks in practice and the wellbeing of staff members during the pandemic. Finally, PRICOV-19 aimed to determine which practice characteristics and health care system features are associated with safe, effective, patient-centered, and equitable health care and with the mental wellbeing of the GPs.

### Study design and setting

During the summer of 2020, an international consortium consisting of over 45 research institutes was established, with Ghent University (Belgium) serving as the coordinating institution, to initiate the PRICOV-19 study. This multi-country cross-sectional study aimed to investigate the organization of PCPs during the COVID-19 pandemic, the modifications to task roles, the impact on the wellbeing of healthcare providers, and any differences that could be observed between various types of practices and healthcare systems. The data were collected from 37 European countries and Israel. For Belgium, data collection took place in all three regions: the Flemish Region (FR), the Brussels-Capital Region (BCR), and the Walloon Region (WR). This paper focuses on the Belgian data.

### Measurement

An online self-reported questionnaire was employed to gather data from PCPs. The questionnaire is developed and validated at Ghent University following a five-step procedure [33]. Firstly, based on the research objectives, a scoping literature review informed the first draft of the questionnaire. Secondly, using a Delphi procedure, a panel of five primary health care (PHC) experts and one methodological expert evaluated the validity of the items and the length of the questionnaire, formulated suggestions for changes, and identified missing items. Next, the research team discussed all feedback until it reached consensus, and a second version of the questionnaire was developed. Thirdly, we organized three cognitive interviews with two GPs and one non-GP to check the acceptability of the questionnaire. Furthermore, an online version of the questionnaire was made using the Research Electronic Data Capture (REDCap) platform [34] and pretested in ten participants (both GPs and non-GPs). Fourthly, we used the new questionnaire version in a pilot study among a convenience sample of 159 GP practices in Flanders (Belgium). We selected GP practices from a list of training practices included in the GP training program and via the peer-learning groups of GP trainees. All selected practices received an invitation by email, including a link to the online questionnaire. Also, we introduced the study in the newsletter of the Flemish Society for General Practice. In the fifth development step, the international consortium partners reviewed the questionnaire for acceptability in their country and cultural adaptation. Finally, the research team discussed all suggested changes until it reached a consensus. The final questionnaire included 53 items divided into six sections: patient flow; infection prevention; information processing; communication; collaboration and wellbeing; and practice and participant characteristics. The REDCap platform was used to host the survey [34].

### Sampling and recruitment

Data were collected between November 2020 and December 2021. Belgian practices were recruited between December 2020 and August 2021. A random sample of 1477 practices was drawn based on the list of GPs on the website of the ‘National Institute for the Sickness and Invalidity Insurance’. The random sample was drawn at GP level as lists of practices are not available in Belgium. It was taken into account that only one GP from the same practice was selected. Being qualified as a GP before 1980 was considered an exclusion criterion to exclude retired GPs or GPs seeing only a limited number of patients. The practices of all selected GPs were invited to participate in the study using a standardized procedure, including several attempts of contact via telephone and email. This resulted in the participation of 370 practices (response rate of 25.1%). An additional convenience sample of 134 PCPs was drawn through the professional and personal networks of the research teams involved. Hereof 109 PCPs participated in the study (response rate of 81.3%). Only one survey was completed per practice, usually by a GP.

### Measures

#### Outcome measure

Four survey questions regarding outreach initiatives were selected as the outcome variables in the analyses (Table 1). A recent publication of the international PRICOV-19 consortium regarding the international data on outreach work also used these four items [15]. To determine if these four survey questions captured multiple components related to outreach work, the research team conducted a principal component analysis (PCA) [35]. More specifically, in this study, a PCA was used to determine the outcome variable by identifying the underlying factors that possibly could contribute to the outcome of interest, being ‘outreach work’. To determine how many factors or principal components to retain, we applied the Kaiser-Guttman rule, which suggests retaining all factors with eigenvalues greater than 1.0. Also, a scree plot was used to visually inspect the eigenvalues and determine the number of factors to retain based on the point at which the curve levels off. Thereafter, the loadings of the variables on each factor was examined to interpret the underlying structure of the data. Variables with high loadings on a given factor were considered to be closely related to that factor, while variables with low loadings may not be well represented by the factor. In this PCA, the scree plot of the eigenvalues indicated that only one factor with a clear eigenvalue exceeding 1 should be retained, and all four questions related to outreach work had high loadings on this one factor (See Appendix 1). A reliability analysis of a mean scale based on the four questions of the COVID-19 scale demonstrated very good internal consistency, with a Cronbach’s α value of 0.735.

**Table 1:** Survey questions and their answer options that were the basis for the outcome variable

Survey question	Answer options
Since the COVID-19 pandemic, a list was compiled from the EMR^(a)^ for at least one group of patients with a chronic disorder (e.g. all patients taking methotrexate and needing to be seen).	□ No □ Yes Missing value: no answer, I do not know
Since the COVID-19 pandemic, this practice has contacted patients with a chronic condition who needed follow-up care.	□ No □ Yes Missing value: no answer, I do not know
Since the COVID-19 pandemic, this practice has contacted psychologically vulnerable patients.	□ No □ Yes Missing value: no answer, I do not know
Since the COVID-19 pandemic, this practice has contacted patients with previous problems of domestic violence or with a problematic child-rearing situation.	□ No □ Yes Missing value: no answer, I do not know

^(a)^*EMR* electronic medical records

Next, a composite variable representing “outreach work” was constructed. Because of the additive effect of all four items, a count variable was constructed. This count could range from 0 to 4. This count variable was recoded into three categories: 0 means no outreach work (count 0), 1 means moderate outreach work (count 1 to 2), 2 means strong outreach work (count 3 to 4). Cases having a missing value (i.e. ‘no answer’ or answered ‘I do not know’) for one of the four items were excluded.

#### Independent variables

Four practice characteristics, two patient population characteristics and an area deprivation index were used as independent variables.

##### Practice characteristics

The four practice covariates included practice type (solo, duo, or group practice based on the number of GPs in practice), being a teaching practice for GP trainees (yes or no), presence of a nurse or a nurse assistant (yes or no), presence of a social worker or health promotor (yes or no). To avoid multicollinearity issues, only the most clinical relevant parameters were modelled.

##### Patient population characteristics

In the survey’s ‘practice characteristics’ section, respondents were asked to what extent they felt their patient population was below, approximately at, or above the average of practices in their country in terms of treating patients with chronic conditions, patients over the age of 70, patients with limited or low health literacy, patients with a migration background with difficulty speaking the local language, patients with financial problems, patients with a psychiatric vulnerability, and patients with little social support or limited informal care. There was also an option for respondents to answer ‘I do not know’. The method of questioning for patient population characteristics in the PRICOV-19 survey is based on the QUALICOPC survey [36]. Because of high inter-relatedness, a factor analysis was done which revealed two components, describing medical complexity and social vulnerability of the practice’s patient population. These two components were retained in the model as two count variables. The medical complexity of the practice population could range from count 0 to 2 (Table 2). The social vulnerability of the practice population could range from count 0 to 5 (Table 3). The latter was recoded into a categorical variable with three ordinal categories: 0 meaning no social vulnerability (count 0), 1 meaning moderate social vulnerability (count 1 to 2) and 2 meaning strong social vulnerability (count 3 to 5) of the practice’s patient population.

**Table 2:** Composition of the ‘medical complexity’ variable

Medical complexity of the practice patient population	
0	Below or approximately the average patients with chronic conditions and patients over the age of 70
1	More than average patients with chronic conditions or patients over the age of 70
2	More than average patients with chronic conditions and patients over the age of 70

**Table 3:** Composition of the ‘social vulnerability’ variable

Social vulnerability of the practice patient population *The items underneath get a score of 1 if they are present above the average in the practice’s patient population*	
Patients with limited or low health literacy	0 Below or approximately the average 1 Above the average
Patients with a migration background with difficulty speaking the local language	0 Below or approximately the average 1 Above the average
Patients with financial problems	0 Below or approximately the average 1 Above the average
Patients with a psychiatric vulnerability	0 Below or approximately the average 1 Above the average
Patients with little social support or limited informal care	0 Below or approximately the average 1 Above the average

Count score of 0 ➔ Social vulnerability 0 (no)

Count score of 1 to 2 ➔ Social vulnerability 1 (moderate)

Count score of 3 to 5 ➔ Social vulnerability 2 (strong)

##### Area deprivation index

A level of area deprivation was assigned to each Belgian municipality [37]. Four variables (population density, average income per capita, percentage inhabitants with migration background and percentage unemployed) were combined into one score by calculating a weighted mean of the component variables by municipality, using a principal component analysis. This score, the area deprivation index was shifted and rescaled to obtain a score ranging from 0 to 100.

Data from the ‘Vlaamse Arbeidsrekening’ of 2018 were used to derive unemployment information for all Belgian municipalities [38]. This was measured as the percentage of the unemployed population between 15 and 64 years old. The Belgian statistical office, STATBEL, provided information on average income per capita [39] for 2019. In addition, STATBEL data provided information on population density [39] and the percentage of inhabitants with a migration background [39] in 2021.

### Data analysis

Baseline characteristics of participating PCPs were analyzed using descriptive statistics (Table 4). Frequencies and percentages were used to describe the outcome variable (Table 5).

**Table 4:** Description of the practice characteristics of the participating Belgian primary care practices and comparison between the Belgian regions: descriptive statistics and chi-square tests

**Characteristics of the PCPs**^a^				
			**N**	**%**
Number of PCPs			462	100%
Practice type	Solo		169	36.6%
	Duo		92	19.9%
	Group		199	43.1%
Multidisciplinary	Yes		138	29.9%
	No		309	66.9%
GP^b^ trainee teaching practice	Yes		194	42.0%
	No		263	56.9%
Payment system	Fee for service		415	89.8%
	Capitation		43	9.3%
Nurse or nurse assistant	Yes		92	19.9%
	No		370	80.1%
Social worker or health promotor	Yes		38	8.2%
	No		424	91.8%
**Characteristics of the PCP’s patient population**				
			**N**	**%**
Medical complexity	None		296	64.1%
	Moderate		74	16.0%
	Strong		92	19.9%
Social vulnerability	None		291	63.0%
	Moderate		96	20.8%
	Strong		75	16.2%
**Characteristics of the PCP’s area deprivation**				
	**Median**	**IQR**^c^	**Minimum**	**Maximum**
Are deprivation index	17.07	10.12; 29.53	0	100

^a^*PCP* Primary care practice

^b^*GP* General practitioner

^c^*IQR* Interquartile range

**Table 5:** Distribution of the amount of outreach work in this study’s primary care practices (*n* = 462)

	Number	Percent
No outreach work	201	43.5
Moderate outreach work	176	38.1
Strong outreach work	85	18.4

Ordinal logistic regression analyses were performed to predict whether PCPs did outreach work. Various associations were taken into account. Odds ratios and 95% confidence interval (CI) were reported. The criterion of statistical significance (two-fold, p) was determined at 0.05. In the case of post-hoc tests, reported confidence intervals and *p*-values are corrected for multiplicity (Holm procedure). The proportional odds assumption is met. Table 6 shows the adjusted model.

**Table 6:** Results of ordinal logistic regression analysis of potential associations with outreach work in primary care practice

Outreach work			
Associated factors	Odds Ratios	95% CI	***p***
Intercept no outreach versus moderate and strong outreach	2.12	1.31 to 3.44	0.002
Intercept no and moderate outreach versus strong outreach	17.14	9.79 to 30.00	< 0.001
Duo versus solo practices	1.15	0.67 to 1.97	0.600
Group versus solo practices	2.21	1.36 to 3.62	0.001
Presence of a GP trainee	1.35	0.89 to 2.06	0.163
Presence of a nurse or nurse assistant	1.86	1.07 to 3.24	0.028
Presence of a social worker or health promotor	2.87	1.24 to 6.86	0.015
Moderate versus no social vulnerability	3.01	1.28 to 7.19	0.013
Strong versus no social vulnerability	2.30	0.74 to 7.08	0.148
Moderate versus no medical complexity	0.78	0.39 to 1.51	0.459
Strong versus no medical complexity	1.16	0.66 to 2.03	0.609
Area Deprivation Index	1.02	1.00 to 1.03	0.016
Social vulnerability 1 * medical complexity 1	0.32	0.08 to 1.24	0.111
Social vulnerability 2 * medical complexity 1	6.99	1.45 to 41.99	0.022
Social vulnerability 1 * medical complexity 2	0.32	0.10 to 0.99	0.050
Social vulnerability 2 * medical complexity 2	0.57	0.13 to 2.44	0.454
Social vulnerability 1 * Area Deprivation Index	0.97	0.94 to 0.99	0.017
Social vulnerability 2 * Area Deprivation Index	0.98	0.95 to 1.01	0.165

Observations 454

Missing data were assessed during the preliminary analysis. The missing value analysis showed that none of the variables of interest had more than 5% of missing values. Furthermore, we created dummy variables for the variables having some missing data (1 = missing, 0 = observed) and we ran t-tests between the dummy variable (of the variable with missing data) and the other variables in the data set to see if the missingness on this variable was related to the values of other variables. This was not the case, so missing data were probably missing completely at random. To proceed with the data, we omitted those cases with the missing data and analyze the remaining data. This approach is known as the complete case analysis or listwise deletion.

Statistical analysis was performed using SPSS software (version 28.0 SPSS IBM Corp., Armonk, N.Y., USA) and R software (version 4.2.1 R Foundation for Statistical Computing, Vienna, Austria).

### Ethical approval

The study was conducted according to the guidelines of the Declaration of Helsinki. The Research Ethics Committee of Ghent University Hospital approved the protocol of the PRICOV-19 study and Belgian data collection (BC-07617). All participants gave informed consent.

## Results

### Description of the participating primary care practices

The characteristics of the 462 Belgian practices are shown in Table 4. Two hundred seventy-two practices were located in the FR (58.9%), 144 (31.2%) in the WR, and 45 (9.7%) in the BCR.

### The statistical model

The distribution of the amount of outreach work is shown in Table 5. Two hundred and one practices did no outreach work (43.5%), 176 (38.1%) did moderate outreach work, and 85 (18.4%) did strong outreach work during the COVID-19 pandemic.

### Practice type based on the number of GPs: solo/duo/group

After correction for the other variables in the model, practice type was positively associated with outreach (χ^2^(2) = 11.82, *p* = 0.003). Post hoc tests revealed that groups were more likely to exert outreach than solos (OR 2.21, 95% CI 1.22 to 4.02, *p* = 0.004) and duos (OR 1.92, 95% CI 1.02 to 3.59, *p* = 0.026). However, we couldn’t find a statistically significant difference between duos and solos (*p* = 0.6, see Table 7 of the Appendix 2 and Additional file 1 for more details).

### GP trainees

Practices with GP trainees have 1.35 (95% CI 0.89 to 2.06, *p* = 0.163) times the odds of exerting more outreach compared to practices without GP trainees. This result is not statistically significant.

### Nurse or nurse assistant

Practices with a nurse (assistant) have 1.86 (95% CI 1.07 to 3.24, *p* = 0.028) times the odds of exerting more outreach compared to practices without a nurse (assistant).

### Social worker and/or health promotor

Practices with a social worker or a health promotor or both have 2.87 (95% CI 1.24 to 6.86, *p* = 0.015) times the odds of exerting more outreach compared to practices without these disciplines.

### The social vulnerability factor

The effect of social vulnerability (SV) of the patient population on outreach work is dependent on the level of medical complexity (MC) of the patient population and the area deprivation index (ADI).

**Fig. 1:**
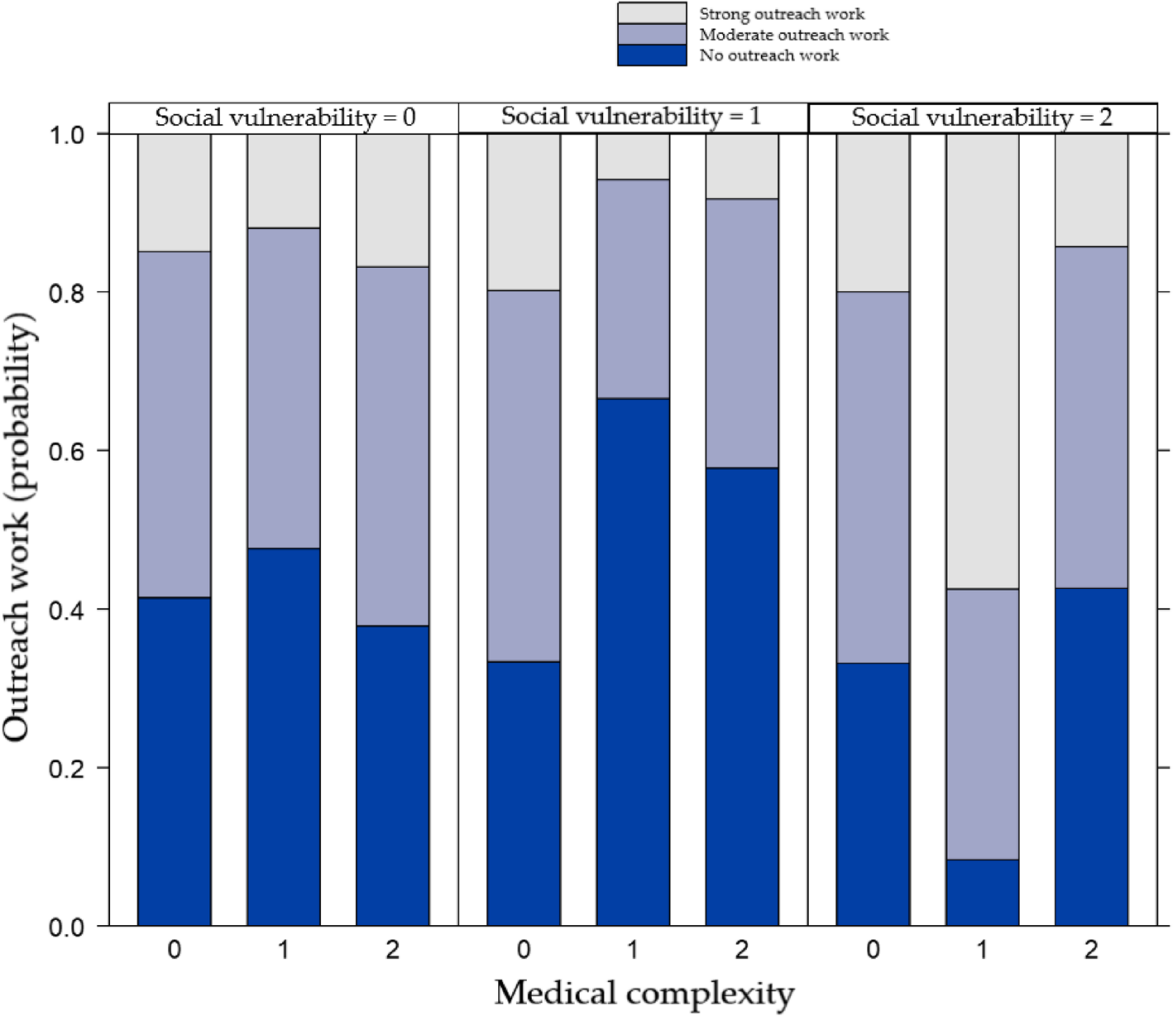
Effect plot of the interaction term social vulnerability * medical complexity

The association of the medical complexity with outreach is dependent on the level of social vulnerability (interaction effect χ^2^(4) = 14.401, *p* = 0.006). At low levels of social vulnerability, there is no apparent association with medical complexity. However, at high levels of social vulnerability, the odds of exerting more outreach are higher when the medical complexity is at moderate levels. Yet, the odds are again lower when the levels of medical complexity further increase to high levels (Fig. 1). Odds ratios, confidence intervals and *p*-values can be found in Table 8 of the Appendix 2 and in Additional file 1.

**Fig. 2:**
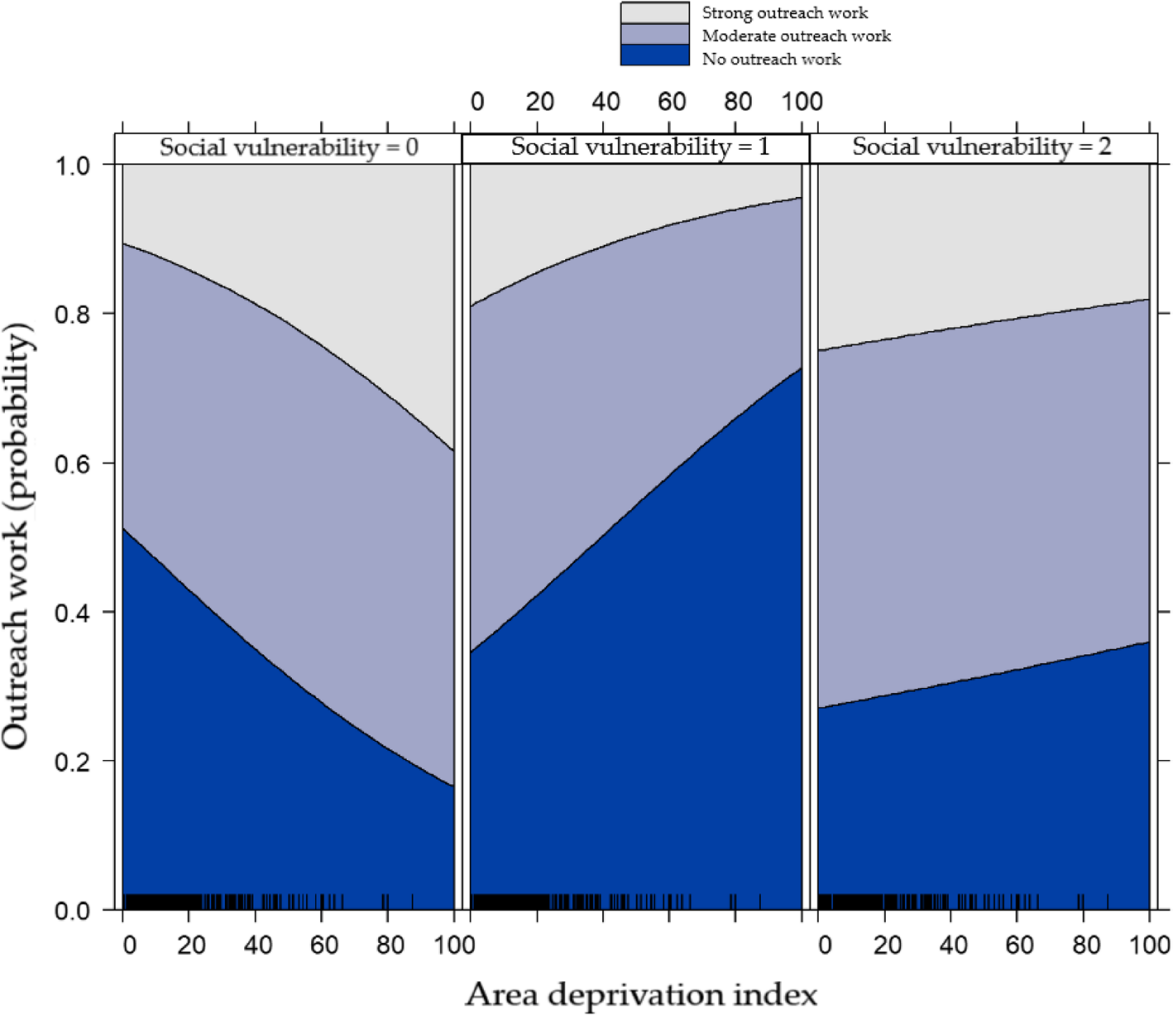
Effect plot of the interaction term social vulnerability * area deprivation index

Area Deprivation Index was positively associated with GP Outreach for GPs with no socially vulnerable populations (OR 1.017, 95% CI 1.003 to 1.031, *p* = 0.015). However, we could not find an association for GPs with moderate (OR 0.984, 95% CI 0.96 to 1.007, *p* = 0.176) and high (OR 0.996, 95% CI 0.971 to 1.023, *p* = 0.754) socially vulnerable populations (Fig. 2).

## Discussion

### Summary of findings

First, the factors significantly associated with outreach work in PCPs are group practice type, the presence of a nurse (assistant) and the presence of a social worker or health promotor. Second, the extent of outreach work done by a PCP is significantly associated with the social vulnerability of the practice’s patient population. This social vulnerability factor, affecting outreach work, differed with the level of medical complexity of the practice’s patient population and with the level of deprivation of the municipality where the practice is situated. At high levels of social vulnerability moderate medical complexity was associated with more outreach work compared to low and high medical complexity. PCPs perform significantly more outreach work when situated in more deprived areas, if the PCP has a low level of socially vulnerable patients. However, we could not find this association for PCPs with moderate and high socially vulnerable populations.

This study is one of the first to examine factors associated with facilitating outreach work in PCPs. As such, this study aimed to generate hypotheses on which factors enable PCPs to perform outreach work to vulnerable populations. The finding that group practices are more likely to engage in outreach work compared to solo and duo practices is not consistent with previous research, as this factor has not yet been explored in published studies on outreach work. However, the debate over the optimal practice size and type in relation to the quality of primary care is ongoing, with limited evidence supporting an association between practice size and quality of care in primary care [40, 41]. According to a Canadian study in 2016 [42], practice type serves as a proxy for various organizational characteristics that may have different associations with various care processes. The study found that the presence of nurses with expanded roles acted as a mediator on the relationship between practice size and patients’ experience of care, preventive services, and unmet needs. As in this study, the presence of a nurse (assistant), social worker or health promotor was significantly associated with more outreach work. Our findings are consistent with previous evidence that emphasizes the importance of having dedicated personnel for the organization of outreach work [43]. Several studies have also shown that non-GP staff involved in outreach work mostly have a background in nursing or social work [44–46].

Several prior studies have documented the positive associations between serving as a training practice for GPs and characteristics of high-quality care in practice organization, chronic care and preventive services [47, 48]. However, in this study, the association between training practices and outreach initiatives was not significant (OR = 1.35, *p* = 0.163).

In socioeconomic deprived areas levels of multimorbidity and social complexity are higher than in less deprived areas. Primary care professionals often encounter difficulties in managing the complex healthcare needs of patients residing in socioeconomically deprived areas, as noted in previous studies [49, 50]. Nevertheless, primary care professionals generally hold a positive attitude towards working with disadvantaged patients and perceive their interactions with them as fulfilling, since they feel they play a crucial role in monitoring their physical, psychological, and social well-being and appreciate their trust [51, 52]. Nonetheless, when their patient population’s overall level of social vulnerability surpasses a certain threshold, they tend to develop a rather negative perception, as poor outcomes and demanding attitudes make it more challenging to provide effective care. As a result, their motivation to devote energy to this patient group diminishes [49, 50]. This contrast is reflected in the present study. Our hypotheses, that PCPs would perform more outreach work when situated in more deprived areas, can only be confirmed for PCPs with a low level of socially vulnerable patients. When the amount of social vulnerability becomes too high, we see the opposite: a decrease in performed outreach work when area deprivation increases. Similar findings apply to the interaction between medical complexity and social vulnerability of PCPs’ patient populations. More specifically, the model presented in this study shows a decrease in outreach work when the medical complexity of the patient population increases. This trend is most pronounced at high levels of social vulnerability of the patient population. This confirms again the hypothesis that GPs can only provide high-quality healthcare when the burden of complex patients is not passing a certain limit [36].

In both policy and research, there has been a recent focus on resilience strategies, such as having control over work organization in order to prevent burnout among GPs [53]. It has been shown that working within a multidisciplinary team that includes colleagues such as nurses or social workers/health promotors provides the necessary support structure to help maintain resilience among GPs [49, 53, 54]. This interprofessional team environment, by allowing for shared tasks, responsibilities, and decision-making, could ease the burden on individual practitioners and allow them to effectively perform outreach work in these challenging times.

### Strengths and limitations

Globally, experts have already stressed the lack of research on the position of primary care during the COVID-19 pandemic [10, 11]. This study provided an answer regarding Belgium based on 462 PCPs. According to earlier studies in primary care [55, 56], response rates of 25.1 and 81.3% for randomized and convenience sampling methods were reasonable for Belgium. Furthermore, the sample composition among the regions corresponded to the actual distribution of the number of GPs in Belgium (IMA-AIM, 2021), which supports sample representativeness.

To our knowledge, this study is one of the first to examine factors associated with facilitating outreach work in PCPs. Given the need for a more careful consideration of the concept of outreach and a better theoretical understanding of outreach approaches [5], this study adds to this demand. However, also a few limitations should be noted.

Firstly, data were collected through an online self-reported survey, so interpretations of the results should be formulated with awareness of the risk of social and professional desirability, which may negatively influence the truthfulness of the answers. The researchers have no insight into the actual practice organization and outreach initiatives that were organized.

Secondly, only one survey is completed per PCP as described in the study protocol, thanks to the close collaboration among the research teams involved. It implies that the truthfulness of the answers also relied on the familiarity of the participating staff member with the practice processes and procedures. However, the function of the participating staff member was not considered in the analyses. Data collection took place from December 2020 until August 2021. This period encompassed three large waves of the COVID-19 pandemic in Belgium, implying that the timing might have affected the study results. Therefore, the results only demonstrated a snapshot of the practice organization during COVID-19. Consequently, making any statements about possible permanent changes in Belgian practices’ practice organization or quality policy is impossible. It follows the suggestion to set up longitudinal studies to research possible changes in the organization of PCPs.

Thirdly, due to the small sample size, problems of overfitting could have occurred. Overfitting refers to the situation where a statistical model captures the noise or random fluctuations in the data rather than the underlying pattern or relationship. When a model is overfit, it performs well on the specific dataset it was trained on but doesn’t generalize well to new, unseen data [57]. In this context of a small sample size, overfitting implies the risk that the model might have learned too much from the specific data points without accurately representing the broader trends or patterns in the entire population.

Fourthly, to ensure participant anonymity in data processing in accordance with the GDPR regulation, the data used to retrieve which practices came from which sample were removed during the transfer of the dataset from Redcap. Due to the relatively low participation rate and the large difference in participation between the random and convenience samples, there is a risk of selection bias.

Fifthly, caution should be exercised when interpreting the composition of the PCPs’ patient population vulnerability. As an item of the PRICOV-19 survey, respondents were requested to provide an estimate of the proportion of certain vulnerable population groups in their practice in comparison to the average practices within their country. However, in order to do so, adequate background knowledge of the patient population in both their own practice and other practices throughout the country is necessary. This may prove to be challenging, as general practitioners, for instance, may tend to overestimate their patients’ income status [58, 59]. As such, there could have been a response bias when respondents were asked to assess whether their practice context was below/above average.

Finally, in this study, we present the area deprivation index and its associated aggregated data, while acknowledging some limitations. To start with, it is important to note that the index assumes internal homogeneity within areas, which may not always reflect reality. For instance, municipalities with a mix of high and low deprivation households may obtain an intermediate ranking score. Furthermore, the use of the area deprivation index renders this study susceptible to the ecological fallacy, which arises from the assumption that inferences can be drawn about individual patterns based on observed group patterns [60, 61]. This assumption may not always hold true, as patterns at the municipal level may differ from those at the individual level. Therefore, we exercise caution in drawing conclusions at the individual level and instead focus on deprivation in areas rather than individuals. Lastly, it is crucial to underscore that there is no singular, definitive definition of deprivation and that there are multiple ways of measuring its value [37]. In this study, an index of area deprivation was assigned to each Belgian municipality. This area deprivation index was defined as a weighted mean of four variables (population density, average income per capita, percentage inhabitants with migration background and percentage unemployed). This definition allows us to gain insights into the spectrum of area deprivation with municipalities ranked from least to most deprived. However, if a different definition with different composing variables -which is off course also dependent on the availability of population data - were to be used, it could potentially have led to different outcomes.

### Implications for practice and research

Primary care professionals play a crucial role in reaching out to vulnerable populations during and after the COVID-19 pandemic, placing them at the center of the healthcare system. However, they face numerous challenges in fulfilling this role. The PRICOV-19 study addressed a gap in current knowledge and fulfilled the need for comprehensive research on the organization of outreach work in primary healthcare during the COVID-19 pandemic [10, 33]. The findings of this study can be used to inform policymakers when developing primary healthcare interventions to reach out to vulnerable populations. These implications extend across various tiers of Belgian governance, encompassing local and national levels, with potential applicability in international contexts within PCPs, considering the diverse needs of vulnerable populations and healthcare infrastructures. Regarding research dissemination, these findings present value in their integration into policy reports or position statements, aiming to inform policymakers and offer recommendations based on empirical evidence. Firstly, it is important to invest in interprofessional collaboration in PCPs, and in expanding responsibilities to different disciplines, such as nurses and social workers. With this, special attention should be paid to very vulnerable regions. Consequently, evaluations should be made to ensure that sufficient support can be provided in vulnerable regions to minimize the task overload for PCPs in these regions. Moreover, interprofessional education and continuous professional training should pay sufficient attention to community-oriented care and should provide training in strategies for PCPs to do outreach work.

Further research is needed to elaborate on how different types of outreach activities can be implemented in PCPs, considering interprofessional education and collaborative practices, in order to organize task shifting to nurses and/or social workers/health promotors, amongst other healthcare professions.

## Conclusions

This study represents one of the earliest attempts to investigate the factors that contribute to facilitating outreach work in PCP. In this study, outreach work in PCPs during the COVID-19 pandemic was facilitated by the group type cooperation of GPs and by the support of other primary care professionals (referring to the involvement of at least one staff member from the disciplines of nursing, social work or health promotion). Also, the extent of outreach work done by a PCP is significantly associated with the social vulnerability of the practice’s patient population. This social vulnerability factor, affecting outreach work, differed with the level of medical complexity of the practice’s patient population and with the level of deprivation of the municipality where the practice was situated. The results suggest that improving the effectiveness of outreach efforts in PCPs requires addressing organizational factors at the practice level. This applies in particular to PCPs having a more socially vulnerable patient population. These findings can be used to inform policymakers on how to support GPs and their PCPs when developing primary healthcare interventions to reach out to vulnerable populations. Further research should focus on elaborating different types of outreach activities, considering task shifting to nurses and/or social workers/health promotors.

### Supplementary Information

**Additional file 1.** BMC PC - Supplementary tables.

## Figures and Tables

**Fig. 3:**
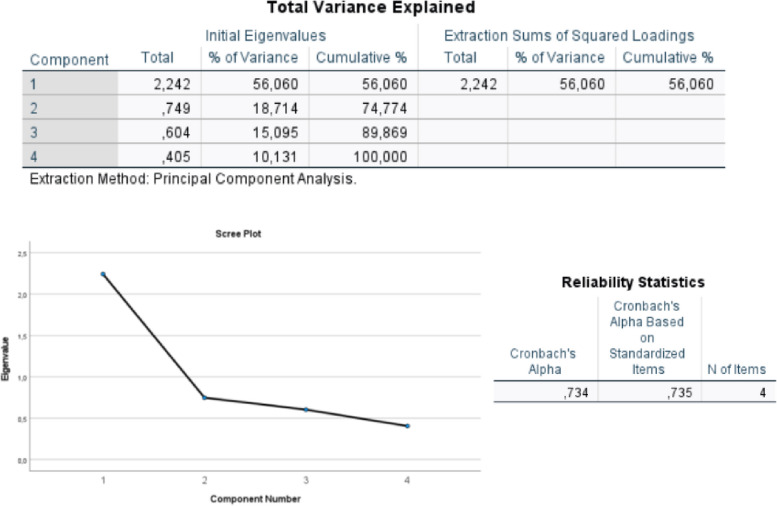
Principal component analysis, scree plot and reliability analysis for the four outreach-related items of the outcome variable

## Data Availability

All data are centrally stored on the server of Ghent University (Belgium). All data was anonymized at Ghent University, and all raw data that could lead to the identification of the respondents was permanently removed. Reasonable request is required to access non-identifiable data by users who are external to the PRICOV-19 consortium. Access will be subject to a data transfer agreement and following approval from the principal investigator of the PRICOV-19 study.

## Appendix 2

**Table 7:** Ordinal logistic regression post-hoc tests for PCPs’ group type

		95% CI		
Contrast	OR	lower	upper	p
Solo versus Duo	0.866	0.450	1.667	0.600
Solo versus Group	0.452	0.249	0.819	0.004
Duo versus Group	0.521	0.278	0.977	0.026

**Table 8:** Ordinal logistic regression post-hoc tests for the social vulnerability (SV) * medical complexity (MC) interaction term

		95% CI		
Contrast	OR	lower	upper	p
SV 0 MC 0 - SV 1 MC 0	0.708	0.277	1.808	1.000
SV 0 MC 0 - SV 2 MC 0	0.701	0.221	2.222	1.000
SV 0 MC 0 - SV 0 MC 1	1.290	0.430	3.864	1.000
SV 0 MC 0 - SV 1 MC 1	2.815	0.443	17.896	1.000
SV 0 MC 0 - SV 2 MC 1	0.129	0.012	1.377	0.188
SV 0 MC 0 - SV 0 MC 2	0.863	0.345	2.161	1.000
SV 0 MC 0 - SV 1 MC 2	1.935	0.437	8.565	1.000
SV 0 MC 0 - SV 2 MC 2	1.053	0.130	8.563	1.000
SV 1 MC 0 - SV 2 MC 0	0.991	0.261	3.761	1.000
SV 1 MC 0 - SV 0 MC 1	1.822	0.505	6.571	1.000
SV 1 MC 0 - SV 1 MC 1	3.978	0.553	28.605	0.783
SV 1 MC 0 - SV 2 MC 1	0.183	0.016	2.142	0.819
SV 1 MC 0 - SV 0 MC 2	1.220	0.387	3.843	1.000
SV 1 MC 0 - SV 1 MC 2	2.733	0.528	14.161	1.000
SV 1 MC 0 - SV 2 MC 2	1.488	0.164	13.472	1.000
SV 2 MC 0 - SV 0 MC 1	1.839	0.436	7.755	1.000
SV 2 MC 0 - SV 1 MC 1	4.014	0.504	31.939	0.869
SV 2 MC 0 - SV 2 MC 1	0.184	0.016	2.189	0.819
SV 2 MC 0 - SV 0 MC 2	1.231	0.326	4.648	1.000
SV 2 MC 0 - SV 1 MC 2	2.758	0.473	16.080	1.000
SV 2 MC 0 - SV 2 MC 2	1.502	0.171	13.205	1.000
SV 0 MC 1 - SV 1 MC 1	2.183	0.283	16.833	1.000
SV 0 MC 1 - SV 2 MC 1	0.100	0.008	1.233	0.115
SV 0 MC 1 - SV 0 MC 2	0.670	0.187	2.397	1.000
SV 0 MC 1 - SV 1 MC 2	1.500	0.267	8.432	1.000
SV 0 MC 1 - SV 2 MC 2	0.817	0.084	7.914	1.000
SV 1 MC 1 - SV 2 MC 1	0.046	0.002	0.869	0.029
SV 1 MC 1 - SV 0 MC 2	0.307	0.044	2.162	1.000
SV 1 MC 1 - SV 1 MC 2	0.687	0.072	6.593	1.000
SV 1 MC 1 - SV 2 MC 2	0.374	0.025	5.665	1.000
SV 2 MC 1 - SV 0 MC 2	6.672	0.571	77.998	0.435
SV 2 MC 1 - SV 1 MC 2	14.950	0.978	228.527	0.053
SV 2 MC 1 - SV 2 MC 2	8.141	0.385	172.194	0.819
SV 0 MC 2 - SV 1 MC 2	2.241	0.443	11.334	1.000
SV 0 MC 2 - SV 2 MC 2	1.220	0.135	10.990	1.000
SV 1 MC 2 - SV 2 MC 2	0.545	0.045	6.556	1.000

## Abbreviations

GP: General practitioner
PC: Primary care
PCP: Primary care practice
FR: Flemish Region
BCR: Brussels-Capital Region
WR: Walloon Region
PHC: Primary heath care
PCA: Principal component analysis
EMR: Electronic medical record
CI: Confidence interval
IQR: Interquartile range
SV: Social vulnerability
MC: Medical complexity
ADI: Area deprivation index
