# Supplementary material for: Outreach work in Belgian primary care practices during COVID-19: results from the cross-sectional PRICOV-19 study
Source: BMC Prim Care. 2024 Apr 3;24(Suppl 1):283. doi: 10.1186/s12875-024-02323-6 (PMC10988793; doi:10.1186/s12875-024-02323-6)
Supplement: Supplementary file 1 — Additional file 1. BMC PC - Supplementary tables. [file 12875_2024_2323_MOESM1_ESM.docx]

Table S1: Ordinal logistic regression post-hoc tests for GP practices' group size

|  |  | **95% CI** | |  |
| --- | --- | --- | --- | --- |
| **Contrast** | **OR** | **lower** | **upper** | **p** |
| Solo versus Duo | 0.866 | 0.450 | 1.667 | 0.600 |
| Solo versus Group | 0.452 | 0.249 | 0.819 | 0.004 |
| Duo versus Group | 0.521 | 0.278 | 0.977 | 0.026 |

Table S2: Ordinal logistic regression post-hoc tests for the social vulnerability (SV) * medical complexity (MC) interaction term

|  |  | **95% CI** | |  |
| --- | --- | --- | --- | --- |
| **Contrast** | **OR** | **lower** | **upper** | **p** |
| SV 0 MC 0 - SV 1 MC 0 | 0.708 | 0.277 | 1.808 | 1.000 |
| SV 0 MC 0 - SV 2 MC 0 | 0.701 | 0.221 | 2.222 | 1.000 |
| SV 0 MC 0 - SV 0 MC 1 | 1.290 | 0.430 | 3.864 | 1.000 |
| SV 0 MC 0 - SV 1 MC 1 | 2.815 | 0.443 | 17.896 | 1.000 |
| SV 0 MC 0 - SV 2 MC 1 | 0.129 | 0.012 | 1.377 | 0.188 |
| SV 0 MC 0 - SV 0 MC 2 | 0.863 | 0.345 | 2.161 | 1.000 |
| SV 0 MC 0 - SV 1 MC 2 | 1.935 | 0.437 | 8.565 | 1.000 |
| SV 0 MC 0 - SV 2 MC 2 | 1.053 | 0.130 | 8.563 | 1.000 |
| SV 1 MC 0 - SV 2 MC 0 | 0.991 | 0.261 | 3.761 | 1.000 |
| SV 1 MC 0 - SV 0 MC 1 | 1.822 | 0.505 | 6.571 | 1.000 |
| SV 1 MC 0 - SV 1 MC 1 | 3.978 | 0.553 | 28.605 | 0.783 |
| SV 1 MC 0 - SV 2 MC 1 | 0.183 | 0.016 | 2.142 | 0.819 |
| SV 1 MC 0 - SV 0 MC 2 | 1.220 | 0.387 | 3.843 | 1.000 |
| SV 1 MC 0 - SV 1 MC 2 | 2.733 | 0.528 | 14.161 | 1.000 |
| SV 1 MC 0 - SV 2 MC 2 | 1.488 | 0.164 | 13.472 | 1.000 |
| SV 2 MC 0 - SV 0 MC 1 | 1.839 | 0.436 | 7.755 | 1.000 |
| SV 2 MC 0 - SV 1 MC 1 | 4.014 | 0.504 | 31.939 | 0.869 |
| SV 2 MC 0 - SV 2 MC 1 | 0.184 | 0.016 | 2.189 | 0.819 |
| SV 2 MC 0 - SV 0 MC 2 | 1.231 | 0.326 | 4.648 | 1.000 |
| SV 2 MC 0 - SV 1 MC 2 | 2.758 | 0.473 | 16.080 | 1.000 |
| SV 2 MC 0 - SV 2 MC 2 | 1.502 | 0.171 | 13.205 | 1.000 |
| SV 0 MC 1 - SV 1 MC 1 | 2.183 | 0.283 | 16.833 | 1.000 |
| SV 0 MC 1 - SV 2 MC 1 | 0.100 | 0.008 | 1.233 | 0.115 |
| SV 0 MC 1 - SV 0 MC 2 | 0.670 | 0.187 | 2.397 | 1.000 |
| SV 0 MC 1 - SV 1 MC 2 | 1.500 | 0.267 | 8.432 | 1.000 |
| SV 0 MC 1 - SV 2 MC 2 | 0.817 | 0.084 | 7.914 | 1.000 |
| SV 1 MC 1 - SV 2 MC 1 | 0.046 | 0.002 | 0.869 | 0.029 |
| SV 1 MC 1 - SV 0 MC 2 | 0.307 | 0.044 | 2.162 | 1.000 |
| SV 1 MC 1 - SV 1 MC 2 | 0.687 | 0.072 | 6.593 | 1.000 |
| SV 1 MC 1 - SV 2 MC 2 | 0.374 | 0.025 | 5.665 | 1.000 |
| SV 2 MC 1 - SV 0 MC 2 | 6.672 | 0.571 | 77.998 | 0.435 |
| SV 2 MC 1 - SV 1 MC 2 | 14.950 | 0.978 | 228.527 | 0.053 |
| SV 2 MC 1 - SV 2 MC 2 | 8.141 | 0.385 | 172.194 | 0.819 |
| SV 0 MC 2 - SV 1 MC 2 | 2.241 | 0.443 | 11.334 | 1.000 |
| SV 0 MC 2 - SV 2 MC 2 | 1.220 | 0.135 | 10.990 | 1.000 |
| SV 1 MC 2 - SV 2 MC 2 | 0.545 | 0.045 | 6.556 | 1.000 |
